# Supplementary material for: The presynaptic ribbon maintains vesicle populations at the hair cell afferent fiber synapse
Source: eLife. 2018 Jan 12;7:e30241. doi: 10.7554/eLife.30241 (PMC5794257; doi:10.7554/eLife.30241)
Supplement: Transparent reporting form [file elife-30241-transrepform.docx]

***eLife’s* transparent reporting form**

We encourage authors to provide detailed information *within their submission* to facilitate the interpretation and replication of experiments. Authors can upload supporting documentation to indicate the use of appropriate reporting guidelines for health-related research (see [EQUATOR Network](http://www.equator-network.org/%20)), life science research (see the [BioSharing Information Resource](https://biosharing.org/)), or the [ARRIVE guidelines](http://www.plosbiology.org/article/info:doi/10.1371/journal.pbio.1000412) for reporting work involving animal research. Where applicable, authors should refer to any relevant reporting standards documents in this form.

If you have any questions, please consult our Journal Policies and/or contact us: [editorial@elifesciences.org](mailto:editorial@elifesciences.org).

**Sample-size estimation**

- You should state whether an appropriate sample size was computed when the study was being designed
- You should state the statistical method of sample size computation and any required assumptions
- If no explicit power analysis was used, you should describe how you decided what sample (replicate) size (number) to use

Please outline where this information can be found within the submission (e.g., sections or figure legends), or explain why this information doesn’t apply to your submission:

Sample sizes were based on previous experimentation where variance estimates were available for doing power analysis for predicted significance levels. In general, we garner data above the required levels, particularly when exploring new technologies and new phenotypes because power analysis are less useful in these cases. For some experiments though, the technical difficulties often limit the number of quality recordings that we can get in a reasonable time period. The body of work presented here represents 2 years of data collection. Sample sizes, repetitions where needed and statistical tests are presented with the data in the text and figure legends and in the methods section under Data Analysis section. Box plots are used so that all samples are apparent.

**Replicates**

- You should report how often each experiment was performed
- You should include a definition of biological versus technical replication
- The data obtained should be provided and sufficient information should be provided to indicate the number of independent biological and/or technical replicates
- If you encountered any outliers, you should describe how these were handled
- Criteria for exclusion/inclusion of data should be clearly stated
- High-throughput sequence data should be uploaded before submission, with a private link for reviewers provided (these are available from both GEO and ArrayExpress)

Please outline where this information can be found within the submission (e.g., sections or figure legends), or explain why this information doesn’t apply to your submission:

The main text, figure legends and methods section presents all sample sizes. As electrophysiology data, each cell represents one animal, cell number and animal number are the same. For immunocytochemistry and TEM data, animal numbers as well as cell number and synapse numbers are presented in the text.

**Statistical reporting**

- Statistical analysis methods should be described and justified
- Raw data should be presented in figures whenever informative to do so (typically when N per group is less than 10)
- For each experiment, you should identify the statistical tests used, exact values of N, definitions of center, methods of multiple test correction, and dispersion and precision measures (e.g., mean, median, SD, SEM, confidence intervals; and, for the major substantive results, a measure of effect size (e.g., Pearson's r, Cohen's d)
- Report exact p-values wherever possible alongside the summary statistics and 95% confidence intervals. These should be reported for all key questions and not only when the p-value is less than 0.05.

Please outline where this information can be found within the submission (e.g., sections or figure legends), or explain why this information doesn’t apply to your submission:

Figure 1

I,J,D lines 101-104

G lines 146-150

Legend for figure 1 I,J,K,L Lines 785-797

Figure 2

Legends provides sample size and statistical tests (along with Methods section on Data Analysis),

Fig 3,4

lines 204-207, 211-213

Legend provides animal number, cell numbers, image numbers, synapse numbers as well as statistical comparisons and p values.

Fig 5

A,B lines 225-226

I, lines 230-231

J lines 295-6

Figure legends provides n values as well as statistical comparisons

Fig 6

B 247-254

E 259-263

Figure legends provide sample sizes as well as statistical comparisons.

Figure 7

Figure and legends contains all sample size and statistical information.

Fig 8

E lines 309-311

F lines 312-315

H 318-320

K 325-327

M 330-332

The figure legend contains the sample sizes as well as all statistical tests

Fig 9

D 354-356

F 349-352

G,H lines 361-362

The figure legend contains all of the statistical tests as well as the sample sizes for each plot.

(For large datasets, or papers with a very large number of statistical tests, you may upload a single table file with tests, Ns, etc., with reference to sections in the manuscript.)

**Group allocation**

- Indicate how samples were allocated into experimental groups (in the case of clinical studies, please specify allocation to treatment method); if randomization was used, please also state if restricted randomization was applied
- Indicate if masking was used during group allocation, data collection and/or data analysis

Please outline where this information can be found within the submission (e.g., sections or figure legends), or explain why this information doesn’t apply to your submission:

Data are separated into WT and RBE KO/KO groups based on genotyping. Data were collected blind for each experimentalist. Data were revealed after data collection to ensure we were populating both groups appropriately (success rates are very low for many of the recordings that we do)

**Additional data files (“source data”)**

- We encourage you to upload relevant additional data files, such as numerical data that are represented as a graph in a figure, or as a summary table
- Where provided, these should be in the most useful format, and they can be uploaded as “Source data” files linked to a main figure or table
- Include model definition files including the full list of parameters used
- Include code used for data analysis (e.g., R, MatLab)
- Avoid stating that data files are “available upon request”

Please indicate the figures or tables for which source data files have been provided:

No additional source data is provided. Additional data access can be provided as needed along with publication of the manuscript.
